# Changes in Prevalence and Severity of Domestic Violence During the COVID-19 Pandemic: A Systematic Review

**DOI:** 10.3389/fpsyt.2022.874183

**Published:** 2022-04-13

**Authors:** Freya Thiel, Verena C. S. Büechl, Franciska Rehberg, Amera Mojahed, Judith K. Daniels, Julia Schellong, Susan Garthus-Niegel

**Affiliations:** ^1^Institute and Policlinic of Occupational and Social Medicine, University Hospital Carl Gustav Carus, Faculty of Medicine, Technische Universität Dresden, Dresden, Germany; ^2^Institute for Systems Medicine (ISM) and Faculty of Medicine, MSH Medical School Hamburg, Hamburg, Germany; ^3^Department of Clinical Psychology and Experimental Psychopathology, Faculty of Behavioural and Social Sciences, University of Groningen, Groningen, Netherlands; ^4^Psychologische Hochschule Berlin, Berlin, Germany; ^5^Department of Psychotherapy and Psychosomatic Medicine, Faculty of Medicine, University Hospital Carl Gustav Carus, Technische Universität Dresden, Dresden, Germany; ^6^Department of Child Health and Development, Norwegian Institute of Public Health, Oslo, Norway

**Keywords:** pandemic, domestic, intimate, partner, violence, abuse, COVID-19

## Abstract

**Background:**

To contain the spread of COVID-19, governmental measures were implemented in many countries. Initial evidence suggests that women and men experience increased anger and aggression during COVID-19 lockdowns. Not surprisingly, media reports and initial empirical evidence highlight an increased risk for domestic violence (DV) during the pandemic. Nonetheless, a systematic review of studies utilizing participants' reports of potential changes in DV prevalence and severity during the pandemic as compared to pre-pandemic times is needed.

**Objective:**

To examine empirical, peer-reviewed studies, pertaining to the potential change in prevalence and severity of different types of DV during the COVID-19 pandemic, as reported by study participants.

**Data Sources:**

Electronic EMBASE, MEDLINE, PsycINFO, and CINAHL searches were conducted for the period between 2020 and January 5, 2022. References of eligible studies were integrated by using a snowballing technique.

**Study Selection:**

A total of 22 primary, empirical, peer-reviewed studies published in English or German were included.

**Results:**

Of the 22 studies, 19 were cross-sectional whereas 3 included both pre-pandemic and during pandemic assessments. Data synthesis indicates that severity of all types of DV as well as the prevalence of psychological/emotional and sexual DV increased for a significant number of victims in the general population during the pandemic. Evidence for changes in prevalence regarding economic/financial, physical, and overall DV remains inconclusive. There was considerable between-study variation in reported prevalence depending on region, sample size, assessment time, and measure.

**Conclusions:**

Data synthesis partly supports the previously documented increase in DV. Governmental measures should consider the availability of easily accessible, anonymous resources. Awareness and knowledge regarding DV need to be distributed to improve resources and clinical interventions.

## Introduction

In order to contain the global spread of COVID-19, measures such as social isolation/distancing, quarantine, and stay-at-home orders have been implemented in many countries (1, 2). Although effective in decelerating the spread of COVID-19 (3, 4), these measures also have major social consequences, which may have a substantial impact on mental health, wellbeing, and life satisfaction (5, 6). Empirical research pertaining to mental health during the COVID-19 pandemic indicates increased levels of anxiety, depression, insomnia, and psychological distress (7). Feelings of loneliness resulting from measures such as social isolation or stay-at-home orders, may not only lead to an increase in depressive symptoms (8), but may also impair self-regulation abilities (9), which can lead to dysfunctional behavioral patterns, such as alcohol and drug abuse (10, 11), as well as violent behavior (12). Initial evidence suggests that during the first COVID-19 lockdown in Germany, both women and men experienced increased anger and aggression and tended to direct their anger at others (13). Over the course of the pandemic, media reports have highlighted an alarming increase in rates of domestic violence among intimate partners and against children during lockdown periods (14–16) and web searches related to support for domestic abuse have expanded since the beginning of the pandemic (17).

Domestic violence (DV) is defined as “a pattern of behavior that is used to gain or maintain power and control over an intimate partner in a relationship, a child, another relative or any other household member” (18). DV may affect anyone, regardless of age, gender, ethnic or socioeconomic background, religious or sexual orientation, or type of relationship (18, 19). To this end, DV can also include intimate partner violence (IPV). According to the World Health Organization (WHO), IPV pertains to “any act or behavior within a present or former intimate relationship that causes physical, psychological, or sexual harm” (20). Among others, these behaviors may include (a) psychological/emotional or verbal violence (e.g., insulting, threatening, humiliating), (b) sexual violence (e.g., forced sexual intercourse), (c) physical violence (e.g., beating, kicking), (d) economic/financial violence (20, 21).

Reports on DV or IPV have largely focused on violence committed against women. To this end, it has been documented that globally one in three women will experience physical or sexual violence committed by an intimate partner during her life (20), making IPV the most common form of violence against women. Nonetheless, public, empirical, and clinical attention toward DV or IPV against men has grown. Similar to violence against women, it is estimated that one in four men will experience physical violence by an intimate partner during his life (22–25). As described above, governmental restrictions to slow down the spread of COVID-19, such as social isolation, have been linked to increased anger and aggression (13), which may in turn increase the risk for DV victimization and/or perpetration. Finally, a recent review documents that both social and geographic isolation represent crucial risk factors for IPV (1).

Despite the positive effects of governmental restrictions on containment of the virus, these measures also deteriorated conditions for victims of DV, finding them trapped at home with their perpetrators and minimizing their access to social support systems like friends and family outside the abusive relationship (15, 26). Further, stay-at-home orders and lockdowns might make it easier for perpetrators to socially isolate and surveil their victim, which may be used to control intimate partners or family members (26). Thus, during the pandemic, the risk of DV may have increased because of domestic confinement with possible perpetrators, while at the same time access to private and public help resources such as protection services has been limited (2, 15, 26). Regional and societal factors may further impact victims' and perpetrators' access to help resources. For instance, in many settings around the world, patriarchal views of the family, social norms, or geographical distance from professional and private support resources may offer potential explanations for the increased risk of IPV (1, 27–29).

Although worldwide media reports suggest increasing rates of DV over the course of the pandemic (14–16) and initial empirical evidence highlights that social and geographical isolation may augment DV (1), empirical studies pertaining to a potential increase in DV cases or severity during the pandemic had to be designed, conducted, and had to undergo rigorous peer-review processes before publication. Since the global onset of the COVID-19 pandemic in March 2020, the amount of empirical, peer-reviewed studies has grown.

To date, several reviews focusing on a change in DV prevalence are available. First, an initial systematic review of 32 studies published until July 2020 documented evidence for an increase in DV cases, specifically during the first week of COVID-19 lockdowns in various countries. Nonetheless, this review was conducted in the early stages of the pandemic—thus, the majority of included studies reported on police or helpline reports to assess DV prevalence and not all included reports and studies had been peer-reviewed (30). Second, a systematic review and meta-analysis of 18 studies published until January 2021 focused exclusively on administrative/official data (e.g., police records), documenting an increase in DV following stay-at-home orders or lockdown, with the majority of studies stemming from the U.S. (31). Third, a systematic review focused solely on IPV, including 19 studies, eight of which focused on reports by victims and 11 on reports by help professionals (i.e., police officers, DV resource center staff, healthcare providers). Results outlined an increase in the episodes of IPV as reported by victims (i.e., cross-sectional studies) and help professionals (32). Fourth, a systematic review focusing on IPV as well as sexual functioning during the COVID-19 pandemic included 11 cross-sectional studies published until the end of 2020, 5 of which reported on IPV. The authors showed that IPV against women increased during the COVID-19 pandemic (33). Taken together, all prior reviews suggest an increase in DV during the pandemic. It should however be noted that prior reviews were limited by the timing of literature and it can be assumed that additional literature has been published since. Further, initial research primarily focused on administrative/official reports to assess a potential change in DV during the COVID-19 pandemic. Nevertheless, initial studies focusing on administrative/official reports may reflect changes in help-seeking behavior rather than changes in prevalence, highlighting the importance for empirical studies assessing participants.

We therefore set forth to examine empirical, peer-reviewed studies reporting on original participant data regarding a change in the prevalence and/or severity of DV over the course of the pandemic as compared to pre-pandemic times. Given the acute nature of the topic and the time needed to plan, conduct, and publish relevant data, we expected the majority of studies to have employed cross-sectional designs. Nonetheless, we also expected initial evidence from longitudinal studies or those with repeated pre-pandemic and during pandemic assessments to be available by the time of the current literature search.

## Materials and Methods

### Search Strategy

In order to examine the research question of whether there was a change in DV prevalence and/or severity during the COVID-19 pandemic as compared to pre-pandemic times, we followed the PRSIMA (34) approach: Electronic EMBASE, MEDLINE, PsycINFO, and CINAHL searches were conducted from 2020 to January 5, 2022 to identify research articles for inclusion in this review. Separate searches for each primary database combined terms relating to DV and the COVID-19 pandemic, applying the Boolean operators (AND) and (OR), accordingly. For MEDLINE, PsycINFO, and CINAHL searches, we used the search string “TI (domestic OR intimate OR interpersonal OR partner OR marital OR couple OR relationship) AND TI (violence or abuse) AND TI (covid^*^ OR pandemic OR corona)”. For EMBASE, the string was adapted to “((domestic OR interpersonal OR intimate OR partner OR marital OR couple OR relationship) AND (violence OR abuse) AND (covid OR pandemic OR corona)).ti.”. Additionally, references of eligible studies were integrated by using a snowballing technique.

### Eligibility Criteria

For inclusion in this review, we considered primary, peer-reviewed, empirical studies pertaining to a potential change in DV prevalence and/or severity during the COVID-19 pandemic as reported by participants, published in English or German. Studies examining participant-reported violence in a domestic context during the pandemic, including different age groups, genders, and any form of intimate relationship (e.g., intimate partner, relationship, marital or couple violence, violence against children in the household) were incorporated. Thus, studies utilizing official records (e.g., police, helpline, or hospital records) without participant assessment were excluded in order to focus specifically on the potential change in DV prevalence rather than a change in help-seeking behavior. Empirical quantitative studies, such as cross-sectional, longitudinal, and clinical studies, published in peer-reviewed journals were included. Qualitative studies, conference abstracts, case studies, and dissertations/theses with a peer-reviewed published version were excluded.

### Data Collection Process

All studies identified through the database searches were imported into the systematic review tool Rayyan QRCI (35). Titles and abstracts were screened by two reviewers (VCSB and FR). Studies which did not meet eligibility criteria were excluded. In case of any uncertainties, a third reviewer (FT) was consulted. Subsequently, full texts were reviewed by the same reviewers as above (VCSB and FR) and screened for final inclusion in the current review. Again, a third reviewer (FT) was consulted in case of insecurities. Included studies were then retained for data extraction.

### Risk of Bias (Quality) Assessment

Studies identified for inclusion in the current review were assessed for risk of bias using the JBI critical appraisal checklist for prevalence studies (36). It includes nine appraisal criteria pertaining to the appropriateness of a study's (1) target population (i.e., sample frame addresses target population), (2) recruitment method (i.e., appropriate to recruit representative sample), (3) sample size (i.e., power calculation provided), (4) description of subjects and setting (i.e., sufficient detail on sample and setting), (5) data analyses (i.e., sufficient coverage of all subgroup samples), (6) measurement validity (i.e., validated measure used to assess DV), (7) measurement reliability (i.e., DV measured in same way for all participants), (8) statistical analyses (i.e., significance test for change in DV prevalence/severity), and (9) response rate. The full checklist and a detailed description of appraisal criteria are available at https://jbi.global/critical-appraisal-tools. After a pilot trial on one included study to ensure feasibility of the JBI checklist for the current purpose, each study was assessed for risk of bias by two independent reviewers (FT and VCSB/FR). Initial inter-rater agreement was high (93%) and disagreements were discussed to reach consensus. Of the nine checklist criteria, appropriateness of the sample size as well as measurement validity and reliability were considered particularly relevant for the current review and thus defined as major domains. Overall, we considered a study to present low risk of bias if at least five of the JBI checklist criteria were fulfilled, including at least one of the three major domains.

### Data Synthesis

Results were synthesized narratively and in tabular form. Studies on DV prevalence can be expected to exhibit high heterogeneity pertaining to target population as well as conceptualization and assessment of violence. We therefore did not conduct any quantitative analyses for this review. Data from identified studies were tabulated in a data extraction form developed by FT and VCSB. With the help of AM, data pertaining to author and year of publication, country, setting (e.g., clinical or population-based) and study period (i.e., time point of COVID-19 pandemic), study design, sample size and characteristics (e.g., final sample, target population, age, gender), measure used to assess DV (e.g., validated measure, self-generated questions), direction of DV (i.e., victimization, perpetration), DV prevalence estimates, and type of DV (i.e., psychological/emotional or verbal, sexual, physical, economic/financial) were extracted. Further, results from risk of bias assessments were visualized and synthesized in tabular form.

## Results

### Description of Studies

**Figure 1:**
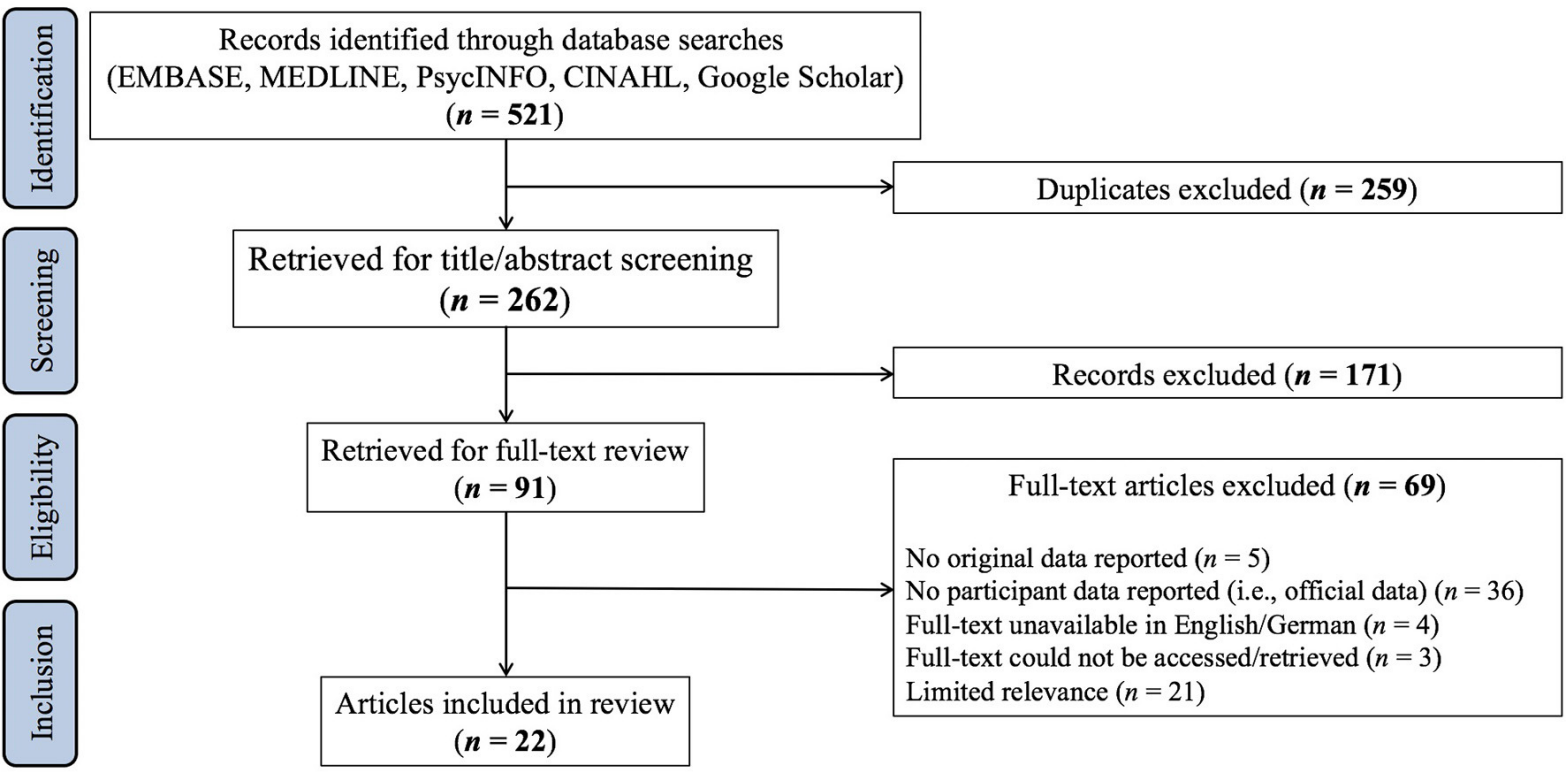
Study selection process.

Electronic EMBASE, MEDLINE, PsycINFO, and CINAHL searches revealed a total of 521 studies. After exclusion of duplicates, titles and abstracts of 262 studies were screened. Based on title/abstract screening, 171 studies were discarded. The remaining 91 studies were retained for full-text screening. Based on full-text screening, 69 studies were excluded because they did not fulfill the eligibility criteria outlined above. Hence, the screening process resulted in the identification and inclusion of 22 studies (13, 37–57). An overview of the study selection process is provided in Figure 1.

### Characteristics of Included Studies

All 22 studies were written in English and published between October 2020 and December 2021, with *n* = 3 published in 2020 and *n* = 19 published in 2021. The studies originated from various countries, with the majority coming from the U.S. (*n* = 4), followed by India (*n* = 3), Germany (*n* = 2), and Bangladesh (*n* = 2). Further studies in this review were conducted in Austria, the Czech Republic, Egypt, Ethiopia, Iraq, Jordan, Nigeria, Peru, Saudi Arabia, Switzerland, and Tunisia. Ten studies included only females (37, 41–43, 46–49, 51, 54), 10 further studies included both female and male participants (13, 39, 44, 45, 50, 52, 53, 55–57), and two studies assessed DV in males only (38, 40). Without exception, all studies reported on DV against adults, with only one study further reporting on violence against children (55). Most studies were cross-sectional (*n* = 19) (13, 37–54), while only few longitudinal studies or studies with repeated pre-pandemic and during pandemic assessments were identified (*n* = 3) (55–57) (see Table 1).

**Table 1:** Study characteristics.

**References**	**Country**	**Study period**	**Study design**	**Sample**	**Type of DV and DV measure**	**Prevalence of DV**	**Change in DV**
Abujilban et al. (46)	Jordan	04/2020 During lockdown	Cross-sectional study Online survey	*n* = 215 pregnant women Age: *M* = 29, *SD* = 4	Psychological IPV Physical IPV Sexual IPV World Health Organization's domestic violence questionnaire screening tool (DVQST)	Pre-lockdown: Psychological violence: 65% Physical violence: 31% Sexual violence: 15% During lockdown: Psychological violence: 50% Physical violence: 13% Sexual violence: 11%	During lockdown: Statistically significant lower mean DVQST scores Decrease of IPV: Psychological Violence: 15% Physical Violence: 49% Sexual Violence: 4%
Alharbi et al. (47)	Saudi Arabia	03-06/2020 During lockdown	Cross-sectional study Online survey	General population *n* = 1,901 married women Saudi: 95% Age: 30–40 yrs: 45%	Physical IPV Psychological IPV Sexual IPV World Health Organization (WHO) multi-country instrument	Pre-lockdown IPV: 25% During lockdown IPV: 17% Among those reporting IPV since the pandemic: Physical IPV: 38% Psychological IPV: 88% Sexual IPV: 17% Multiple forms: 96% Out of those experienced multiple forms of violence: Type of DV (*n* = 301) Physical violence: 38% Psychological violence: 88% Sexual violence: 17%	Overall decrease in IPV: 9% *Among those reporting multiple forms of IPV, frequency and intensity of IPV since COVID-19 Increased: 40% Unchanged: 43% Decreased: 13% Stopped: 4%
Chiaramonte et al. (57)	USA	7–12+ months after COVID-19 1–6 months before COVID-19; 7–12 months before COVID-19; 13–18 months before COVID-19; 19–24+ months before COVID-19	Longitudinal study In-person interview data from an ongoing longitudinal study	7–12+ months after COVID-19 *n* = 406 1–6 months before COVID-19 *n* = 375 7–12 months before COVID-19 *n* = 369 13–18 months before COVID-19 *n* = 359 19–24+ months before COVID-19 *n* = 306 Participants who had sought service from DV agencies	Physical abuse Emotional abuse Stalking Sexual abuse Economic abuse Composite Abuse Scale (CAS) The revised scale of economic abuse (SEA2)	NR	Before the onset of COVID-19: Decrease of all forms of abuse After the onset of COVID-19: No significant differences of all forms of abuse
El-Nimr et al. (49)	Egypt	04-06/2020 During lockdown	Cross-sectional study Online survey	General population; *n* = 490 Arab women living with husband Women from: Saudi Arabia, United Arab Emirates, Kuwait, Qatar, Oman, Yemen, Palestine, Iraq, Jordon, Syria, Egypt, Libya, Sudan, Morocco Age: *M* = 35, *SD* = 8	Verbal IPV Psychological IPV Physical IPV Sexual IPV Financial IPV Self-generated 21-item questionnaire	Pre-lockdown: Any IPV: 40% Verbal: 27% Psychological: 20% Physical: 7% Sexual: 9% Financial: 11% During lockdown: Any IPV: 47% Verbal: 27% Psychological: 27% Physical: 13% Sexual: 14% Financial: 13%	Significant increase of IPV: Any IPV: 7% Psychological: 6% Physical: 6% Sexual: 5% Verbal: no significant change Financial: no significant change
Hamadani et al. (48)	Bangladesh	05-06/2020 During lockdown	Cross-sectional study Phone-based interview	General population; *n* = 2,174 mothers of children enrolled in the Benefits and risks of iron interventions in children (BRISC) trial, living with their husbands Age: *M* = 24, *SD* = 5	Emotional IPV (insults, humiliation, intimidation) Physical IPV Sexual IPV Self-generated questions based on WHO multicountry survey tool	Emotional violence: (Insults: 20%, humiliation: 9%, intimidation: 14%) Physical violence: 7% Sexual violence: 3%	*Reported increased IPV: Emotional: (Insults: 68%, humiliation: 66%, intimidation: 69%) Physical: 56% Sexual: 51%
Indu et al. (54)	India	07/2020-01/2021 Following lifting of the lockdown in June 2020	Cross-sectional study Interview survey; conducted in-person	General population; *n* = 209 Married women residing in the village Panchayat, India during the lockdown period Age: *M* = 36, *SD* = 8	IPV perpetrated by husband over past 12 months Domestic violence questionnaire (DVQ)	Mild DV: 20% (score <5) Severe DV: 6% (score ≥ 5) At least one DV item: 26%	*Out of those reporting at least on DV item: Onset of DV during the lockdown: 11% Worsening of DV during the lockdown: 6%
Jetelina et al. (45)	USA	04/2020 Early stages of the pandemic	Cross-sectional study Online survey	General population; *n* = 1.730 Female: 59% Age: *M* = 42, *SD* = 13	Verbal IPV Psychological IPV Physical IPV Sexual IPV Extended Hurt; insulted, Threated and Screams (E-HITS) construct	Any IPV: 18% Out of those reporting IPV: Verbal violence: insulting: 97%; or screaming: 86% Psychological violence: threaten: 9% Physical violence: 8% Sexual violence: 16%	*Since COVID-19 outbreak: Out of those screened positive for victimization: Victimization remained stable: 54% Physical: 23% Insult: 54% Screams: 53% Threaten: 44% Sexual: 47% Victimization worsened: 17% Physical: 27%
							Insult: 17% Screams: 16% Threaten: 20% Sexual: 28% Victimization improved: 30% Physical: 50% Insult: 30% Screams: 31% Threaten: 36% Sexual: 26%
Jung et al. (13)	Germany	04/2020 During lockdown	Cross-sectional study Online survey	General population; *n* = 3,545 Female: 83% Age: *M* = 40, *SD* = 12	DV Verbal violence Physical violence Sexual violence Self-generated questionnaire	Any DV violence: 5% Out of those reporting DV violence: Verbal violence: female: 98%; male 100% Physical violence: female: 38%; male: 63% Sexual violence: female 27%; male 50%	During lockdown: *Out of those reporting interpersonal violence: Reported experiencing increased levels: Verbal violence: female: 77%; male: 78% Physical violence: female: 15%; male: 21% Sexual violence: female: 3%; male: 0%
Kliem et al. (55)	Germany	01-03/2016 02-03/2021 During lockdown (02-03/2021)	Comparison of two cross-sectional in-person omnibus surveys	General population; 2016: *n* = 1,317 Mean age participants with partner: 50 yrs; with children: 40 yrs; Mean age youngest child in household: 8 yrs 2021: *n* = 1,005 Mean age participants with partner: 52 yrs; with children: 40 yrs; Mean age youngest child in household: 8 yrs)	Physical partner violence (victimization and perpetration) Psychological and physical violence directed at children in past 12 months Three modules of Family Maltreatment Measure	2016 Physical partner violence: Victim: female: 9%; male: 9% Perpetrator: female: 7%; male: 9% Violence directed at youngest child: Physical: female: 20%; male: 22% Psychological: female: 10%; male: 9% 2021 Physical partner violence: Victim: female: 9%; male: 7% Perpetrator: female: 8%; male: 6% Violence directed at youngest child: Physical: female: 16%; male: 18% Psychological: female: 7%; male: 11%	No significant changes in 12-month prevalence rates found (2016 vs. 2021)
Lampe et al. (44)	Austria	04/2020 During lockdown	Cross-sectional study Phone-based interviews	*n* = 67 Female: 76% Age: *M* = 49, *SD* = 14	Verbal violence Physical violence Psychological violence Hurt-Insult-Threaten-Scream (HITS) scale	Scored above the cut-off for DV within the last 2 weeks: 22% Verbal violence (screamed at, insulted): 55% Physical violence: 20% Psychological violence (threats): 0%	Compared to pre-lockdown, overall reported violence significantly decreased in at-risk sample but remained stable in control sample
Mahmood et al. (42)	Iraq	06/2020 After lockdown period (March-May 2020)	Cross-sectional study Online survey	General population; *n* = 346 married women Age: *M* = 38, *SD* = 9	Emotional IPV Physical IPV Sexual IPV Self-generated questionnaire	Pre-lockdown: Any IPV: 32% Emotional IPV: 30% Physical IPV: 13% Sexual IPV: 10% Serious physical injury: 6% During lockdown: Any IPV: 39% Emotional IPV: 35% Physical IPV: 18% Sexual IPV: 11% Serious physical injury: 7%	Significant increase of DV during lockdown: Any violence: 7% Emotional violence: 5% Physical violence: 5% Forcing sexual intercourse: 3%
Ojeahere et al. (39)	Nigeria	05/2020 During lockdown	Cross-sectional study Online survey	General population; *n* = 474 coupled men and women; Female (68%) Age: *M* = 41, *SD* = 8	Physical IPV Financial IPV Emotional IPV Sexual IPV Self-generated questionnaire	Pre-lockdown: Overall IPV prevalence: 14% Physical violence: 3% Financial violence: 3% Emotional violence: 11% Sexual violence: 4% During lockdown: Overall IPV prevalence: 7% Physical violence: 1% Financial violence: 3% Emotional violence: 4% Sexual violence: 2%	Significant decrease of IPV: Overall IPV prevalence: 7% Physical violence: 2% Emotional violence: 7% Sexual violence: 2% Financial violence: no significant change
Pattojoshi et al. (43)	India	05/2020 During lockdown	Cross-sectional study Online survey	General population; *n* = 560 women married for at least 6 months Age: *M* = 38, *SD* = 9	Physical IPV Sexual IPV Verbal IPV Emotional IPV Self-generated 17-item questionnaire for spousal violence	IPV before lockdown: 14% Current violence: 18% Among those reporting current IPV: Physical IPV: 35% Sexual IPV: 11% Verbal IPV: 65% Emotional IPV: 44%	*Among those reporting IPV before lockdown: Increase since lockdown: 78% First IPV during lockdown: 5% Overall increase of IPV: 33%
Plášilová et al. (51)	Czech Republic	11/2020 Retrospective report: T0: 3 months before COVID-19 T1: first wave (03-05/2020) T2: second wave (10-11/2020)	Cross-sectional study Online survey	General population; *n* = 429 women living with a partner for at least 3 months before COVID-19 Age: *M* = 48, *SD* = 16	6 types of IPV: Economic, social, emotional, physical, sexual-psychological, sexual-physical Adapted shortened 6-item questionnaire based on WHO IPV interview (scored 0–2, total IPV score 0–12)	T0: IPV *M* = 0.6, *SD* = 1.7 T1: IPV *M* = 0.5, *SD* = 1.7 T2: IPV *M* = 0.5 *SD* = 1.6	Small, significant decrease in mean IPV incidence from pre-pandemic to first and second COVID-19 waves
Porter et al. (52)	Peru	08-10/2020 During lockdown	Cross-sectional study List randomization experiment Phone-based survey	General population; *n* = 1,992 young adults between 18 and 26 years old from the Young Lives study Female: 50% Age: *M* = 20	Physical DV Self-generated questions	NR	Significant increase in physical DV during lockdown: 8%
Rashid Soron et al. (50)	Bangladesh	08-09/2020 During lockdown	Cross-sectional study Online survey	General population; *n* = 136 Female: 74% Age: *M* = 24, *SD* = 5	Physical DV Psychological DV Sexual DV Economical DV Self-generated questionnaire	Overall DV prevalence: 37% Among those experiencing DV: Pre-lockdown: Physical: 19% Psychological: 65% Sexual: 2% Economic: 6% During lockdown: Physical: 11% Psychological: 68% Sexual: 3% Economic: 16%	*Increase of DV: Psychological: 3% Sexual: 1% Economical: 10% *Decrease of DV: Physical: 8%
Sediri et al. (41)	Tunisia	04-05/2020 During lockdown	Cross-sectional study Online survey	General population; *n* = 751 women Age: *M* = 37, *SD* = 8	Psychological IPV Economic IPV Physical IPV Self-generated questionnaire	IPV before the lockdown: 4%	Significant increase if IPV during the lockdown DV during the lockdown: 15% Out of those reporting DV during lockdown: Psychological violence: 96% Economic violence: 41% Physical violence: 10%
Sharma and Khokhar (53)	India	04/2020 During lockdown	Cross-sectional study Online survey	General population; *n* = 94 married men and women Female: 59% Age: *M* = 40, *SD* = 10	Verbal violence Physical violence Sexual violence Financial violence Self-generated questionnaire	DV in the past year: 9% Among those reporting DV in past year: Verbal violence: 63% Physical violence: 38% Sexual violence: 25% Financial violence: 25%	*DV during lockdown: 7% Among those reporting DV during lockdown: Increase in DV frequency: 86% Increases in Verbal violence: 57% Physical violence: 29% Sexual violence: 14% Financial violence: 29%
Steinhoff et al. (56)	Switzerland	04–09/2018 and 2020 Prepandemic (In-person interview; 04-09/2018) During lockdown (Online survey 1-3; 04-05/2020) Post-lockdown (Online survey 4; 09/2020)	Longitudinal study 2018: in-person interview 2020: 4 online survey assessments Data from Swiss longitudinal community-representative Zurich project on the social development from childhood to adulthood (z-proso)	General population; *n* = 786 Female: 58%	Physical violence; Adapted conflict tactics scale	Perpetration physical DV: 16% Perpetration physical DV and self-harm: 3%	Significant increase of DV: Perpetration among males from 5% in April 2020 to 10% in late May 2020
Stephenson et al. (38)	USA	04-05/2020 During lockdown	Cross-sectional study Online survey	*n* = 516 Gay, Bisexual, and Other Men who have Sex with Men (GBMSM) The majority of the sample was aged between 25 and 44 years	Emotional IPV Sexual IPV Physical IPV Gay, and bisexual men intimate partner violence (IPV-GBM) scale	During lockdown: Any victimization of IPV: 13% Emotional IPV: 10% Sexual IPV: 2% Physical IPV: 2% Any perpetration of IPV: 6% Emotional IPV: 5% Sexual IPV: 1% Physical IPV: 2%	*Out of those reporting experiencing IPV: First time IPV experience during lockdown: Any IPV: 5% Emotional IPV: 3% Sexual IPV: 2% Physical IPV: 1% First time perpetration of IPV during lockdown: Any IPV: 1% Emotional IPV: 0.6% Sexual IPV: 0.2% Physical IPV: 0.2%
Teshome et al. (37)	Ethiopia	08-11/2020	Cross-sectional study Interview survey; conducted in-person	*n* = 464 pregnant women; prenatal clients from prenatal care clinic of St. Paul's Hospital Millennium Medical College (SPHMMC) Age: *M* = 28, *SD* = 5	Physical IPV Sexual IPV Emotional IPV WHO Multi-Country Study on Women's Health and Domestic Violence Against Women questionnaire	Lifetime IPV exposure: 15% Out of those reporting lifetime IPV: Physical violence: 46% Sexual violence: 29% Emotional violence: 75% Within the year of the interview: 10% Out of those reporting IPV within the year of the interview: Emotional Violence: 77% Sexual Violence: 39% Physical Violence: 32% During current pregnancy: 7% Out of those reporting IPV during current pregnancy: Emotional Violence: 73% Sexual Violence: 49% Physical Violence: 30% Among all women, <2% screened for IPV at the prenatal clinic	*Out of those reporting IPV within the year of the interview (*n* = 44): Increased IPV since COVID-19: 18% Increased physical violence: 25% Increased sexual violence: 25% Increased emotional violence: 50%
Walsh et al. (40)	USA	07-09/2020	Cross-sectional study Online survey	*n* = 214 Gay, Bisexual, and Other Men who have Sex with Men (GBMSM) recruited from previous male couples/HIV-related studies *n* = 102 coupled partners (i.e., *n* = 51 couples) Age: *M* = 36, *SD* = 9	Emotional IPV Physical IPV Sexual IPV Gay and Bisexual Men Intimate Partner Violence scale	Victimization: 7% Perpetration: 7% Both: 8% Emotional IPV: new or continuing victimization: 11% Physical IPV: 4% Sexual IPV: 4% Within coupled partners (*n* = 102): Self-reported IPV victims: *n* = 14; partners agreeing with reported victimization: *n* = 5	*Among self-reported victims (*n* = 32): New or more or frequent IPV experience during pandemic: 47% Among self-reported perpetrators (*n* = 29): Increased IPV behavior during pandemic: 34%

*IPV, Intimate Partner Violence; DV, Domestic Violence; NR, Not Reported*.

* *No statistical significance test reported on this change*.

The majority of the *n* = 19 cross-sectional studies assessed different types of DV. To this end, 12 studies reported on changes in psychological/emotional or verbal DV (13, 37–40, 42, 45, 46, 48–50, 53), 11 on sexual DV (13, 37–39, 42, 45, 46, 48–50, 53), 12 reported on physical DV (13, 37–39, 42, 45, 46, 48–50, 52, 53), and 4 included economic/financial DV (39, 49, 50, 53). One study did not provide a differentiation of DV type (54). All cross-sectional studies included participant report on DV victimization, whereas two studies further included assessment of DV perpetration (38, 40). Most cross-sectional studies investigated DV in the general population (*n* = 14) (13, 39, 41–43, 45, 47–54), while some studies focused on specific samples (*n* = 5) (37, 38, 40, 44, 46). Assessment of DV was heterogeneous regarding the methodological approach—five studies used validated questionnaires [i.e., (Extended-) Hurt, Insult, Threaten, Scream Scale [(E-)HITS]; Composite Abuse Scale Revised Short Form (CASR-SF); Domestic Violence Questionnaire (DVQ); Gay and Bisexual Men Intimate Partner Violence scale (IPV-GBM)] (38, 40, 44, 45, 54), nine studies relied on self-generated questions (13, 39, 41–43, 49, 50, 52, 53), five studies used scales from the “*WHO Multi-Country Study on Women's Health and Domestic Violence Against Women*” (37, 47), the “*World Health Organization's Domestic Violence Questionnaire Screening Tool”* (46), or DV self-generated questionnaires based on questionnaires developed by the WHO (48, 51). Further, the majority utilized online surveys (*n* = 14) (13, 38–43, 45–47, 49–51, 53), whereas some studies conducted in-person (*n* = 2) (37, 54) or telephone interviews (*n* = 3) (44, 48, 52).

Compared to the numerous cross-sectional studies, studies with repeated pre-pandemic and during pandemic assessments were still scarce at the time of literature search for the current review. Three empirical, peer-reviewed studies were identified. Of these, two employed longitudinal designs (56, 57) and one compared two representative population surveys from 2016 to 2021 (55). Two studies utilized samples from the general population in Germany and Switzerland (55, 56), with one solely focusing on perpetration of physical DV but not victimization (56), and the other focusing on victimization and perpetration of physical IPV and perpetration of physical and psychological/emotional violence against children in the household (55). The third study was conducted in the U.S. and focused specifically on DV survivors in precarious or unstable housing conditions (57). While two studies conducted in-person interviews at all measurement points (55, 57), one study supplemented pre-pandemic interviews with data collected *via* online surveys during the pandemic (56).

### Quality of Included Studies

**Figure 2:**
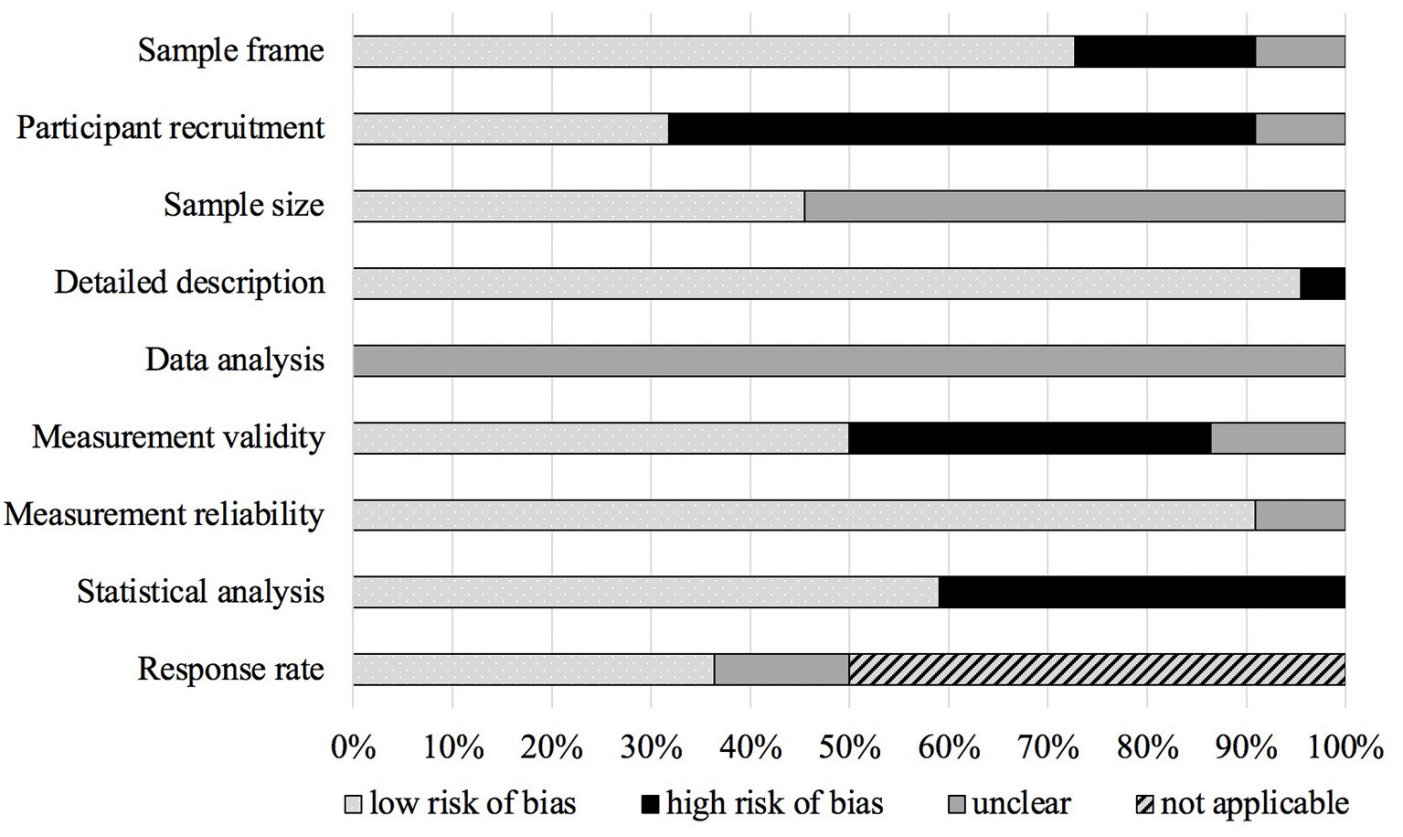
Risk of bias of included studies with percentages of ratings on JBI checklist.

Of the 22 studies, half were rated as having high risk of bias (13, 37, 39, 41–43, 45, 48, 50, 53, 55) and half were rated as having low risk of bias (38, 40, 44, 46, 47, 49, 51, 52, 54, 56, 57). The distribution of ratings on each of the nine JBI checklist criteria (36) can be found in Figure 2. Participant recruitment was rated as holding high risk of bias for 13 of the included studies. This risk of bias mostly pertained to potential selection bias given recruitment for online surveys using snowballing sampling and/or survey distribution *via* various (social) media sites. Sample size was rated as holding low risk of bias only if a power analysis was provided by the original authors and an appropriate sample size was reached. More than half of the included studies (*n* = 12) did not report a power calculation and were consequently rated as “unclear”. Measurement validity was assessed based on the utilization of a generally validated DV measure, without guaranteeing the instrument's validation for use in specific populations or validation of specific translated or adapted versions. Consequently, risk of bias pertaining to measurement validity was rated as low for 11 of the included studies. Although some of the included cross-sectional studies utilizing online surveys reported how many individuals accessed their survey in comparison to the number of completed surveys, an actual response rate cannot be provided—for these studies, the response rate criterion was thus not applicable. Further, because some studies only reported a change in DV prevalence and/or severity in a descriptive manner and did not provide tests of statistical significance for change estimates, 10 of the included studies were rated as holding high risk regarding the statistical analysis criterion. Risk of bias assessment by JBI checklist items for each included study can be found in Supplementary Table S1.

### Domestic Violence During the COVID-19 Pandemic

#### Victimization of Violence

Without exception, all cross-sectional as well as two of the three studies with longitudinal/repeated pre-pandemic and during pandemic assessments reported on changes in DV in the context of victimization (for reports pertaining to DV perpetration, please refer to Section Perpetration of Violence). In the following, we will report results pertaining to changes in each type of DV (i.e., psychological/emotional or verbal, sexual, physical, economic/financial) and changes in overall DV. In each section, we will first present results from studies with longitudinal/repeated pre-pandemic and during pandemic assessments, followed by results from cross-sectional studies. For cross-sectional studies, we will first focus on the change in prevalence and the change in severity in samples from the general population, followed by studies utilizing specific samples. As outlined in the introduction, some studies investigated IPV, which we here conceptualize as a specific type of DV (*n* = 14). We will therefore refer to either IPV or DV depending on the particular focus of the original study.

##### Psychological/Emotional or Verbal Violence

Prevalence of current psychological/emotional or verbal violence varied widely across studies, depending on region, sample size, assessment time, and assessment measure. Only one longitudinal study investigated psychological/emotional violence. Chiaramonte et al. (57) used data from an ongoing longitudinal study in the U.S. to examine the impact of the COVID-19 stay-at-home order (March 15, 2020) on DV survivors who had sought service from DV agencies and were currently in precarious or unstable housing conditions. Five in-person interviews were conducted every 6 months over a 2-year period, assessing psychological/emotional violence *via* the Composite Abuse Scale (CAS). In this specific sample of DV survivors, there was a significant decrease in psychological/emotional DV in the 24 months prior to the onset of the COVID-19 pandemic—thus, since seeking help from a DV agency. No significant changes were found after the onset of the pandemic (57).

Among the *n* = 10 cross-sectional studies reporting on psychological/emotional DV, *n* = 6 studies reported a specific change in psychological/emotional DV prevalence or severity compared to pre-pandemic levels in samples from the general population (39, 42, 45, 48–50), with three studies documenting an increase in overall prevalence and one study documenting a decrease. Two studies reported a significant increase in psychological/emotional DV between 5 and 6% during or after the first lockdown in samples of 346 married women in the Kurdistan region of Iraq (42) and 490 Arab women from 14 different countries (see Table 1) (49). An additional study reported an increase of 3% in a sample of 136 Bangladeshi females and males, but did not report a significance test for this potential increase (50). In contrast, Ojeahere et al. reported a decrease of 7% in a Nigerian sample of 474 female and male participants during the first lockdown using self-generated questions in an online survey (39). All four studies utilized self-generated questions to assess DV *via* online surveys (39, 42, 49, 50).

Regarding a change in severity, two cross-sectional studies specifically reported on changes in psychological/emotional violence among those experiencing DV in samples from the general population (45, 48). To this end, Hamadani et al. differentially examined insults, humiliation, and intimidation as specific types of psychological/emotional IPV *via* telephone interviews in a sample of 2,174 mothers in Bangladesh, recruited from a study in which their children were enrolled. Of those reporting IPV, 68, 66, and 69% reported insults, humiliation, and intimidation to have increased during the first lockdown, respectively (48). Additionally, Jetelina et al. found that among those reporting psychological/emotional IPV in an online survey of 1,730 female and male participants in the U.S., 20% reported violence to have worsened since the COVID-19 outbreak, whereas 36% reported violence to have improved, and 44% reported violence to not have changed (45).

Further, *n* = 4 cross-sectional studies focused on specific populations, namely currently pregnant women in Ethiopia (37) and Jordan (46), as well as gay, bisexual, and other men who have sex with men (GBMSM) in the U.S. (38, 40). Regarding psychological/emotional DV among pregnant women, Abujilban et al. reported a 15% decrease during the first lockdown in an Jordanian sample of 215 pregnant women when using the World Health Organization's Domestic Violence Questionnaire Screening Tool (DVQST) in an online survey (46). Conversely, using in-person interviews, Teshome et al. documented that among those reporting IPV within the last year (i.e., 2020) in their sample of 464 pregnant Ethiopian women, half the women reported psychological/emotional violence to have increased after the COVID-19 outbreak (37). Regarding psychological/emotional DV among gay, bisexual, and other men who have sex with men (GBMSM) in the U.S., using the Gay, and Bisexual Men Intimate Partner Violence Scale in an online survey, Walsh et al. documented that out of those reporting psychological/emotional IPV, 11% reported experiencing new or more frequent psychological/emotional IPV during the pandemic (40). Further, Stephenson et al. report that 1% of the 516 men in their sample indicated having experienced psychological/emotional violence for the first time during the first lockdown (38).

In addition, *n* = 4 cross-sectional studies reported a specific change in verbal DV prevalence to pre-pandemic levels in the general population (13, 45, 49, 53). To this end, two studies indicated verbal DV among 3,545 females and males in Germany (13) and 94 females and males in India (53) who reported experiencing IPV or DV to have increased by 57–78% during the first lockdown. Both studies relied on self-generated questions to assess verbal IPV/DV in online surveys (13, 53). Jetelina et al., however, documented that among those reporting verbal IPV in an online survey of 1,730 female and male participants in the U.S., 17% reported violence to have worsened since the COVID-19 outbreak, whereas 31% reported violence to have improved, and 54% reported violence to not have changed (45). In contrast, El-Nimr et al. found no significant change in verbal IPV during the first lockdown in a sample of 490 Arab women (49). No studies with longitudinal/repeated pre-pandemic and during pandemic assessments of verbal violence were identified.

Taken together, included studies suggest an increase in cases and severity of psychological/emotional DV in the general population during the COVID-19 pandemic. The limited number of studies focusing on specific samples point toward unchanged or even decreased psychological/emotional DV cases, whereas severity of DV may have increased for a significant proportion of victims. Studies pertaining to verbal DV were limited to reports on severity, suggesting verbal DV to have worsened for many victims since the COVID-19 outbreak.

##### Sexual Violence

Only one longitudinal study investigated sexual violence. Chiaramonte et al. (57) used data from an ongoing longitudinal study in the U.S. to examine the impact of the COVID-19 stay-at-home order (March 15, 2020) on DV survivors who had sought service from DV agencies and were currently in precarious or unstable housing conditions (see above). In this specific sample of DV survivors, there was a significant decrease in sexual DV in the 24 months prior to the onset of the COVID-19 pandemic—thus, since seeking help from a DV agency. No significant changes were found after the onset of the pandemic (57).

Among the *n* = 11 cross-sectional studies reporting on sexual DV, *n* = 8 studies reported a specific change in sexual DV prevalence or severity to pre-pandemic levels in samples from the general population (13, 39, 42, 45, 48–50, 53), of which three indicated an increase and one a decrease in overall prevalence. Two studies reported significant overall increases of sexual DV by 3–5% during the first lockdown in samples of Arab women (49) and women in the Kurdistan region of Iraq (42). An additional study reported an increase of 1% in a sample of 136 Bangladeshi females and males, but did not report a significance test for this potential increase (50). In contrast, Ojeahere et al. documented a decrease of 2% in a sample of 474 females and males during the lockdown in Nigeria (39). All four studies utilized self-generated questions to assess sexual DV *via* online surveys (39, 42, 49, 50).

Regarding a change in severity, four studies specifically reported on changes in sexual violence during the first lockdown among those experiencing DV (13, 45, 48, 53). In a sample of 3,545 females and males in Germany, 3% of the women reported sexual violence to have worsened (13). Similarly, in a sample of 94 Indian females and males, 14% of those experiencing DV reported an increase of sexual violence (53). Hamadani et al. reported that out of those experiencing IPV in a sample of 2,174 mothers in Bangladesh, 51% reported sexual violence to have increased (48). Even more specifically, Jetelina et al. documented that among those experiencing sexual IPV in a sample of 1,730 females and males in the U.S., 28% reported violence to have worsened since the COVID-19 outbreak, whereas 26% reported violence to have improved, and 47% reported violence to not have changed (45).

The remaining *n* = 3 cross-sectional studies focused on specific populations, namely currently pregnant women in Ethiopia (37) and Jordan (46), and GBMSM in the U.S. (38). Regarding sexual DV among pregnant women, Abujilban et al. reported a 4% decrease during the first lockdown in a Jordanian sample of 215 pregnant women when using the World Health Organization's Domestic Violence Questionnaire Screening Tool (DVQST) in an online survey (46). Nonetheless, using in-person interviews, Teshome et al. documented that among those reporting IPV within the last year (i.e., 2020) in their sample of 464 pregnant Ethiopian women, 25% reported sexual violence to have increased after the COVID-19 outbreak (37). In the sample of GBMSM men in the U.S., Stephenson et al. documented that 2% indicated having experienced sexual violence for the first time during lockdown (38).

Overall, included studies suggest an increase in cases and severity of sexual DV in the general population during the COVID-19 pandemic. Similar to studies on psychological/emotional violence, the limited number of studies focusing on specific samples point toward unchanged or even decreased sexual DV cases, whereas severity of sexual DV may have increased for a significant proportion of victims.

##### Physical Violence

Two studies with longitudinal/repeated pre-pandemic and during pandemic assessments reported on changes in physical violence. First, Kliem et al. (55) utilized data from in-person interviews between January and March 2016 (i.e., pre-pandemic) and February and March 2021 (i.e., during-pandemic) in representative samples of 1,317 (2016) and 1,005 (2021) participants from the general German population. At both time points, participants reported on physical IPV within the past 12 months. No significant difference between 12-month prevalence from 2016 vs. 2021 were found regarding physical IPV, with the 12-month prevalence remaining stable at around 9% for women and 7–9% for men (55). Second, Chiaramonte et al. (57) used data from an ongoing longitudinal study in the U.S. to examine the impact of the COVID-19 stay-at-home order (March 15, 2020) on DV survivors (see above). In this specific sample of DV survivors, there was a significant decrease in physical DV in the 24 months prior to the onset of the COVID-19 pandemic—thus, since seeking help from a DV agency. No significant changes were found after the onset of the pandemic (57).

Among the *n* = 12 cross-sectional studies reporting on physical DV, *n* = 9 studies reported a specific change in physical DV prevalence to pre-pandemic levels in samples from the general population (13, 39, 42, 45, 48–50, 52, 53), with three studies documenting an increase in overall prevalence and two studies documenting a decrease. Three studies reported significant overall increases of physical DV by 5–8% during the first lockdown in samples of 490 Arab women (49), 346 women in the Kurdistan region of Iraq (42), and 1,992 young Peruvian female and male adults (52). Two studies utilized self-generated questions to assess physical DV *via* online surveys (42, 49), whereas one study conducted phone interviews (52). In contrast, using self-generated questions in an online survey, Ojeahere et al. documented a slight decrease of 2% in a sample of 474 females and males during the lockdown in Nigeria (39). Further, Rashid Soron et al. reported a decrease of 8% in a sample of 136 Bangladeshi females and males, but did not report a significance test for this potential decrease (50).

Regarding a change in severity, four studies specifically reported on changes in physical violence among those experiencing DV (13, 45, 48, 53). Among those reporting DV in a sample of 3,545 females and males in Germany, severity of physical DV increased by almost 15% in females and 21% in males during the first lockdown (13). Similarly, in a sample of 94 Indian females and males, 29% of those experiencing DV reported physical violence to have increased during the first lockdown (53). Hamadani et al. reported higher numbers, documenting that out of those experiencing IPV in a sample of 2,174 mothers in Bangladesh, 56% reported physical violence to have increased during the lockdown using self-generated questions in a phone-based survey (48). Even more specifically, Jetelina et al. documented that among those experiencing physical IPV in a sample of 1,730 females and males in the U.S., 27% reported violence to have worsened since the COVID-19 outbreak, whereas 50% reported violence to have improved, and 23% reported violence to not have changed (45).

The remaining *n* = 3 cross-sectional studies focused on specific populations, namely currently pregnant women in Ethiopia (37) and Jordan (46), and GBMSM in the U.S. (38). Regarding currently pregnant women, Abujilban et al. reported a 49% decrease during the first lockdown in an Jordanian sample of 215 pregnant women when using the World Health Organization's Domestic Violence Questionnaire Screening Tool (DVQST) in an online survey (46). Nonetheless, using in-person interviews, Teshome et al. documented that among those reporting IPV within the last year (i.e., 2020) in their sample of 464 pregnant Ethiopian women, 25% reported physical violence to have increased since the COVID-19 outbreak (37). In the sample of GBMSM in the U.S., Stephenson et al. documented that 1% indicated having experienced physical violence for the first time during the lockdown (38).

Taken together, regarding changes in cases of physical DV in the general population, three cross-sectional studies reported increases, whereas two studies reported decreases during the pandemic. The two studies with longitudinal/repeated pre-pandemic and during pandemic assessments reported no change in cases of physical DV in the general population and among DV survivors. It should however be noted that all studies originated in different countries, making direct comparison difficult. Regarding changes in severity of physical DV, included studies highlight that during the pandemic, physical violence worsened for a significant number of victims. Again, the limited number of studies focusing on specific samples point toward an unchanged or even decreased number of physical DV cases, whereas severity of DV may have increased for a significant proportion of victims.

##### Economic/Financial Violence

Only one longitudinal study investigated economic violence. Chiaramonte et al. (57) used data from an ongoing longitudinal study in the U.S. (see above), assessing economic violence *via* The Revised Scale of Economic Abuse (SEA2). In this specific sample of DV survivors, there was a significant decrease in economic DV in the 24 months prior to the onset of the COVID-19 pandemic—thus, since seeking help from a DV agency. No significant changes were found after the onset of the pandemic (57).

Without exception, all *n* = 4 cross-sectional studies reporting a specific change in economic/financial DV prevalence to pre-pandemic levels utilized samples from the general population (39, 49, 50, 53). Rashid Soron et al. reported a 10% increase during the first lockdown in a sample of 136 Bangladeshi females and males using self-generated questions in an online survey, although no significance test was performed for this potential increase (50). In contrast, also utilizing self-generated questions in online surveys, the two remaining studies did not find any significant change in economic/financial DV during the lockdown in samples of 474 females and males in Nigeria (39) and 490 Arab women (49). Nonetheless, of those experiencing DV in a sample of 94 females and males, 29% reported economic violence to have increased during the first lockdown in India (53).

Overall, three of the four cross-sectional studies as well as the only longitudinal study identified for this review reported no change in economic/financial DV cases during the COVID-19 pandemic in the general population. Nonetheless, for many of those experiencing DV, severity of economic/financial violence may have increased.

##### Changes in Overall Violence

A total of *n* = 14 cross-sectional studies documented changes in overall DV, i.e., regardless of DV type, either through participants' retrospective reports for a time point prior to the pandemic (38–43, 45, 47, 49, 51, 53, 54) or through comparison of cross-sectional data to data collected as part of a prior study (44). Of these, *n* = 10 studies utilized samples from the general population. Four studies reported significant increases in overall DV of 7–33% during the first lockdown in samples of 490 Arab women (49), 346 women in the Kurdistan region of Iraq (42), 751 Tunisian (41), and 560 Indian women (43). All four studies utilized self-generated questions to assess DV *via* online surveys and exclusively focused on violence against women. In contrast, three studies documented decreases in overall DV. To this end, Ojeahere et al. reported a 7% decrease of any type of DV during the first lockdown as compared to pre-lockdown times in a Nigerian sample of 474 females and males using self-generated questions in an online survey (39). Similarly, Alharbi et al. documented an overall 9% decrease of IPV during the first lockdown in a Saudi Arabian sample of 1,901 married women using the WHO multi-country instrument in an online survey (47). Although utilizing mean IPV scores rather than prevalence rates, Plášilová et al. found a small, significant decrease in mean IPV incidence from 3 months prior to the pandemic to measurement time points during the first and second COVID-19 waves in a sample of 429 women in the Czech Republic (51).

Regarding a change in severity, *n* = 4 cross-sectional studies documented changes in those with DV experiences specifically. To this end, Pattojoshi et al. reported that among the 560 women in their sample who experienced IPV before the first lockdown in India, 78% reported an increase in violence since the beginning of the lockdown (43). Similarly, in a sample of 94 females and males, Sharma and Khokhar documented that of those experiencing DV during the lockdown in India, 86% reported increased violence as compared to the time before the pandemic (53). Slightly lower increases were reported by Indu et al. who found that among those having experienced DV perpetrated by their husbands within the previous 12 months in a sample of 209 Indian women, 6% indicated violence to have worsened during the lockdown and 11% reported violence to have begun during the pandemic (54). Even more specifically, Jetelina et al. documented that among those experiencing IPV in a sample of 1,730 females and males in the U.S., 17% reported violence to have worsened since the COVID-19 outbreak, whereas 30% reported violence to have improved, and 54% reported violence to not have changed (45). Further, Alharbi et al. found that among those indicating ever having experienced IPV in a sample of 1,901 married women in Saudi Arabia, 40% reported violence to have increased since the COVID-19 outbreak, whereas 13% reported a decrease, 43% reported no change, and 4% reported violence to have stopped (47).

Four cross-sectional studies focused on specific populations, namely currently pregnant women in Ethiopia (37), GBMSM in the U.S. (38, 40), and participants with a history of DV in Austria (44). Using in-person interviews, Teshome et al. documented that out of those reporting IPV within the last year (i.e., 2020) in their sample of 464 pregnant Ethiopian women, 18% reported experiencing increased violence (37). Two studies investigated IPV in U.S. samples of 516 (38) and 214 (40) GBMSM. Stephenson et al. reported that among self-reported victims of IPV, 5% indicated having experienced IPV for the first time during the first lockdown (38). Walsh et al. documented that among self-reported victims of IPV, 47% reported experiencing new or more frequent IPV since the COVID-19 outbreak (40). Finally, Lampe et al. (44) compared DV during the lockdown in Austria in a sample of female and male participants with (*n* = 34) or without (*n* = 33) prior DV experiences. Those with prior DV experiences reported more DV than those without prior DV experiences. Importantly, while DV remained stable compared to pre-lockdown values for those without prior DV experiences, it decreased in the group with prior DV experiences. Nonetheless, DV during the lockdown remained significantly higher in the group with prior DV experiences (44). No studies with repeated longitudinal/pre-pandemic and during pandemic assessments of overall violence were identified.

Taken together, evidence pertaining to changes in overall DV cases remains inconclusive with four cross-sectional studies reporting increases and three cross-sectional studies reporting decreases. Regarding changes in DV severity however, across different samples from the general population in various countries, 6–86% of those experiencing DV reported violence to have worsened during the first lockdown in their respective country or since the COVID-19 outbreak. Again, the limited number of studies focusing on specific samples does not allow for conclusions regarding changes in the number of overall DV cases, while severity of DV may have increased for a significant proportion of victims.

#### Perpetration of Violence

Besides the focus on victims of DV, *n* = 2 studies with longitudinal/repeated pre-pandemic and during pandemic assessments reported on DV perpetration. First, Steinhoff et al. (56) used interview data from a Swiss longitudinal study to compare DV perpetration in a representative sample of 786 young adults. To this end, pre-pandemic in-person interview reports from 2018 and four during-pandemic online survey measurements between spring and fall 2020 were included. The risk of DV perpetration doubled over the early course of the pandemic from 5% in April 2020 to 10% in May 2020 for men, but no change was observed for women (56). Second, Kliem et al. (55) utilized data from in-person interviews between January and March 2016 (i.e., pre-pandemic) and February and March 2021 (i.e., during-pandemic) in representative samples of 1,317 (2016) and 1,005 (2021) participants from the general German population. At both time points, participants reported on physical IPV perpetration and physical or psychological violence against the youngest child in the household within the past 12 months. No significant difference in the 12-month prevalence from 2016 vs. 2021 were found regarding IPV perpetration or for physical or psychological violence directed against children. IPV 12-month prevalence remained stable with around 6% of women and 6–9% of men reporting IPV perpetration in 2016 and 2021. Similarly, DV directed against children over the past 12 months remained stable with 16–20% of women and 18–22% of men indicating having been physically violent and 7–10% of women and 9–11% of men indicating psychological violence against a child in 2016 and 2021 (55).

Further, *n* = 2 cross-sectional studies examined rates in DV perpetration during the pandemic, both utilizing U.S. samples of GBMSM (38, 40). In their sample of 516 men, Stephenson et al. (38), 6% of participants reported having perpetrated any type of IPV, with emotional IPV being the most common type. Only 1% of men indicated first-time perpetration during the lockdown (38). Reports of perpetration were slightly higher in Walsh et al.'s (40) sample of 214 men, recruited from two previous male couples/HIV-related studies. Overall, 15% reported IPV perpetration, with 7% reporting perpetration but not victimization and 8% reporting both perpetration and victimization. Among the self-reported perpetrators, around a third indicated their behavior to have increased since the COVID-19 outbreak. Interestingly, however, Walsh et al. further documented that among couples within the sample, reports of perpetration and victimization were not always congruent (40).

Overall, the limited number of included studies reporting on DV perpetration does not allow for definite conclusions. Nonetheless, across studies, self-reported perpetration seems to have remained unchanged as compared to pre-pandemic times. The single study documenting perpetration across the pandemic however, indicates that for men, risk of DV perpetration may have increased over time since the COVID-19 outbreak. This finding highlights the need for data from multiple measurement points over the course of the pandemic rather than solely comparing pre-pandemic levels to during-pandemic levels.

## Discussion

The aim of our review was to examine the change in prevalence of domestic violence during the COVID-19 pandemic in empirical, peer-reviewed studies. We opted to only include self-report studies to approximate prevalence rates not biased by help seeking behavior, which in itself might have been altered by the pandemic. Overall, 22 studies were included-−19 were cross-sectional whereas 3 included both pre-pandemic and during pandemic assessments. Of the 22 studies, 17 utilized samples from the general population, while 5 included samples from specific populations [i.e., DV survivors; pregnant women; gay, bisexual, and other men who have sex with men (GBMSM)].

Taken together, these studies suggest (1) an increase in cases and severity of psychological/emotional and sexual DV in the general population, (2) no change in number of economic/financial DV cases in the general population, and (3) an increase in severity of DV of any type for a significant number of victims during the pandemic. Evidence for changes in prevalence regarding verbal DV remains inconclusive because of the limited number of studies reporting on verbal DV. Further, despite a larger number of available studies, evidence for changes in prevalence regarding physical and overall DV remains inconclusive.

As mentioned above, only five of the 22 included studies focused on samples from specific populations, namely DV survivors, pregnant women, and GBMSM. Although it should be assumed that individuals from these three groups would be included in representative samples from the general population, several considerations should be noted. First, although valuable information pertaining to a change in DV severity may be drawn from studies utilizing samples of DV survivors, given the fact that prior DV experience is a risk factor for future DV experiences, a potential change in DV prevalence from pre-pandemic to pandemic times in these samples may not be generalizable to the general nor other populations. Second, we here treated studies on pregnant women as a specific sample because of the additional stress pregnancy and the transition to parenthood may represent for the entire family. To this end, pregnancy-specific factors, such as becoming a first-time parent and the pregnancy being unwanted have been found to put pregnant women at an increased risk for DV victimization (58). Further, violence during pregnancy may have severe adverse consequences for both, the mother and the unborn child. For instance, while physical violence against the pregnant woman may also lead to injuries of the unborn child, implications of maternal mental health complications during pregnancy, potentially resulting from violence victimization, may bear further adverse implications for pregnancy and birth outcomes, as well as child development (59–63). Third, we also treated studies on GBMSM as a specific sample because prior research indicates higher risk for IPV and/or DV among GBMSM than among heterosexual men (64–66). In addition, it has been suggested that sexual minorities may be disproportionately affected by pandemic-related stressors relating to employment, finances, and (mental) health (40). For these reasons, it is noteworthy that only a very limited number of studies on specific (at-risk) groups was available for inclusion in this review. Examinations of other at-risk groups, such as sexual minorities apart from GBMSM and investigations of at-risk samples in different countries is currently still lacking. Thus, changes in DV prevalence and severity in specific (at-risk) groups requires additional scientific attention.

Similarly, it should be highlighted that the majority of included studies reporting on samples from the general population focused on violence against women, with 10 studies exclusively assessing females. Although 10 further studies included both, females and males, there is currently a lack of studies reporting on male victimization. This lack however does not only pertain to DV during the pandemic, but can be pointed out as a gap in the current literature pertaining to DV in general. Additionally, only one of the included studies reported on DV against children. On the one hand, this may be attributable to the current inclusion criterion of solely incorporating studies which presented participant reports. For instance, studies utilizing official/administrative data indicate that DV against children may oftentimes be reported by third parties and that opportunities for third-party observations and report are limited by governmental measures such as social distancing, school closures, and lockdown (67, 68). On the other hand, this may be at least partially explained by the current focus on DV victimization. Only few studies in this review included participant reports regarding DV perpetration. Although not surprising given the topic's sensitive nature and potential biases in self-reports, such as social desirability, examining victimization and perpetration in isolation may not reflect the true complexity and oftentimes bidirectionality of DV, where many individuals may, at least temporarily, be victim and perpetrator rather than one of the two exclusively (69, 70). Nevertheless, a clear picture of DV perpetration and its risk factors is crucial for the development and implementation of resources and (preventive) interventions as well as de-stigmatization of help seeking among perpetrators.

As noted above, at the time of the literature search for this review, only three studies with longitudinal/repeated pre-pandemic and during pandemic assessments were identified. Of these, two utilized samples from the general population in Germany and Switzerland (55, 56), with one solely focusing on perpetration of physical DV but not victimization (56), and the other focusing on victimization and perpetration of physical IPV and perpetration of psychological/emotional and physical violence against children in the household (55). The third study focused specifically on U.S. DV survivors in precarious or unstable housing conditions (57). Without question, more time is required for studies utilizing repeated assessments over time to be conducted and for results to be published. Nonetheless, studies identified for this review highlight a need for data pertaining to prevalence and severity of different types of DV from multiple timepoints prior to the COVID-19 outbreak and over the course of the pandemic with multiple measurements during the pandemic. Repeated assessments over the course of the pandemic are further warranted given different pandemic phases and waves, which in turn may be characterized by differential stressors (71, 72). For instance, the COVID-19 outbreak and immediate governmental measures represented an entirely new and unknown situation for most of the global population, characterized by uncertainty and an immediate increase in stress related to employment and finances for many. Although this initial uncertainty may by now have decreased, long-term adjustment to the pandemic and the ever-changing implications for day-to-day life may vary considerably among individuals given their specific experiences and living conditions. Thus, repeated assessments of DV over the course of the pandemic may offer the opportunity to distinguish between the pandemic's initial stress, potentially resulting in emotional turmoil, in turn increasing the risk for interpersonal aggression, vs. long-term stress, potentially resulting in emotional depletion/depression in turn also increasing the risk for interpersonal aggression (71, 72).

In light of the fast, global spread of COVID-19 and the time needed to design, authorize, and conduct empirical studies, it is not surprising that the majority of studies identified for this review were cross-sectional and utilized online surveys to assess DV. We noted a large between-study heterogeneity regarding study country of origin, sample size, participant inclusion criteria, and/or measure used to assess DV. Taking this into account, results of individual studies should thus be interpreted with caution and may not be generalizable to different regions or samples and are limited regarding the validity of reported changes over time. In addition, few studies reported on self-reported DV perpetration, suggesting that perpetration seems to have remained unchanged as compared to pre-pandemic times.

Putting our results into the context of previous reports using official/administrative data highlights an additional concern. Although studies included in the current review focusing on self-reported DV suggest increased DV experience for a significant amount of people around the world, prior studies utilizing police and helpline call data and formal police reports are not fully congruent with this increase. To this end, several studies have documented decreases in formal DV-related police reports, whereas sharp increases in numbers of DV-related emergency calls to the police and helplines have been documented (73–81). Importantly, the reported reduction in formal police reports may not reflect a decrease in DV prevalence but rather a decrease in reporting DV incidences. Being constrained to the domestic setting and isolation from other social or work contexts due to stay-at home orders or lockdowns may be linked to reduced or altered help-seeking behaviors. Prior research suggests a shift in help-seeking behavior where victims may seek help in acute emergency situations but may not follow through with formal police reporting during stay-at-home orders and lockdown (2). Nonetheless, social isolation may make reporting of DV more difficult given that the perpetrator cannot be separated. Thus, many victims may only have limited or no access to help resources and may further be limited in their ability to participate in research studies and/or to complete online surveys in a safe, unhindered environment. Because this may not be conveyed in crime statistics, empirical studies regarding DV-related help-seeking behavior and potential changes resulting from governmental measures in response to the spread of COVID-19 are needed in order to improve assistance for victims.

The aforementioned changes in help-seeking behavior and restricted or limited resource availability are of particular importance because of the detrimental side effects of DV victimization. For instance, Iob et al. document that half of those experiencing psychological or physical DV reported thoughts pertaining to suicide and self-harm. Alarmingly, during the first U.K. lockdown, a quarter of those experiencing psychological/emotional or physical DV indicated having harmed themselves during the past week (82). Besides the previously documented increase in DV-related homicide during the COVID-19 pandemic (83–85), victims may thus further be at high risk for self-harm and/or suicide, highlighting the crucial need for easily accessible DV resources and (preventive) interventions for both, victims and perpetrators.

### Strengths and Limitations

Several strengths and limitations of the included literature should be acknowledged. It is crucial to highlight the important contributions of the studies included in this review, given the initial reliance on official/administrative records to assess the potential change in DV during the COVID-19 pandemic. Studies included in this review utilized participant reports and may thus more accurately reflect changes in DV prevalence and severity rather than changes in help-seeking behavior. Limitations of included studies pertain to the reliance on cross-sectional designs (viz. introducing potential biases given retrospective self-report) and online surveys (viz. introducing self-selection bias within the sample). Although noted as a limitation in the majority of studies, generalizability of individual results may be limited given concerns regarding sample representativeness of the intended target population. Further, not all studies utilized measures to assess DV which had previously been validated in the language used or for the population investigated. Additionally, the majority of studies focused on DV victimization and only few studies investigated both, victimization and perpetration. Nonetheless, the cultural diversity represented within the identified studies is remarkable, particularly given the timely nature of the topic.

Strengths of the current review are the systematic search for and identification of relevant literature, the systematic processes of data extraction and quality assessment, as well as its focus on participant-reported changes in specific types of DV prevalence and severity estimates and its bi-directionality (i.e., victimization vs. perpetration). Several limitations should be noted. First, given the expectation that studies on DV prevalence tend to exhibit high heterogeneity regarding target population and conceptualization and assessment of violence, we synthesized extracted data narratively and did not conduct any quantitative analyses of reported changes in DV prevalence or severity. Thus, we do not present pooled estimates and our assumption that the considerable variation of changes in prevalence and severity estimates observed may be attributable to between-study variation was not tested. Second, the current review was not pre-registered and no formal protocol was put into writing. Third, although we conducted this systematic review in line with PRISMA guidelines and utilized the JBI checklist for risk of bias assessment, we did not conduct certainty assessments. Fourth, quality assessment presented herein was limited by methodological limitations and lacking information in the original articles. Our risk of bias assessment resulted in the appraisal of half the included studies as presenting high risk. It should therefore be noted that our review may be affected by publication and/or reporting biases.

## Conclusion

In this review, we focused our attention on changes in prevalence and severity of different types of DV during the COVID-19 pandemic. To this end, we examined empirical studies utilizing self-reported participant data, published in peer-reviewed journals. Given the considerable between-study heterogeneity pertaining to region, sample size and characteristics, assessment time, and assessment measure, results of individual studies may not be directly comparable and should be interpreted with caution because of limited generalizability. Overall, our data synthesis of 22 studies indicates increases in cases of psychological/emotional and sexual DV as well as increases in severity of DV of any type for a significant number of victims during the pandemic in the general population. Our findings thus partially support the previously documented increase in DV during stay-at-home orders and lockdown. Nonetheless, evidence for changes in prevalence regarding economic/financial, physical, and overall DV remains inconclusive. Prior research suggests that many victims may only have limited or no access to help resources and that social isolation may make reporting of DV more difficult given that the perpetrator cannot be separated. This highlights an important public and clinical concern, indicating a potential change in help-seeking behavior among victims of DV during the COVID-19 pandemic. Restricted or limited access to help resources and social isolation from friends, family, or co-workers resulting from governmental measures to contain the spread of the virus likely impacts millions of individuals at risk for DV around the world. Governmental measures should thus take into account the availability of easily accessible, anonymous help resources for DV victims and perpetrators, in particular during times of social isolation, stay-at-home orders, and lockdown. Finally, DV awareness and knowledge needs to be distributed in order to improve formal and informal resources as well as (preventive) interventions for both, victims and perpetrators.

## Data Availability Statement

The original contributions presented in the study are included in the article/Supplementary Material, further inquiries can be directed to the corresponding author.

## Author Contributions

FT, VB, and SG-N designed and conceptualized the present study. FT, VB, and FR conducted manuscript screening, data extraction, and risk of bias (quality) assessment. AM aided in data extraction. FT and VB wrote the first draft of the manuscript. SG-N supervised data extraction and drafting of the manuscript. FT, VB, FR, AM, JD, JS, and SG-N contributed to the analysis and interpretation. All authors contributed to manuscript revision, read, and approved the submitted version.

## Funding

The authors received funds for open access publication fees by the Norwegian Institute of Public Health.

## Conflict of Interest

The authors declare that the research was conducted in the absence of any commercial or financial relationships that could be construed as a potential conflict of interest.

## Publisher's Note

All claims expressed in this article are solely those of the authors and do not necessarily represent those of their affiliated organizations, or those of the publisher, the editors and the reviewers. Any product that may be evaluated in this article, or claim that may be made by its manufacturer, is not guaranteed or endorsed by the publisher.
