# Supplementary material for: Changes in Prevalence and Severity of Domestic Violence During the COVID-19 Pandemic: A Systematic Review
Source: Front Psychiatry. 2022 Apr 13;13:874183. doi: 10.3389/fpsyt.2022.874183 (PMC9043461; doi:10.3389/fpsyt.2022.874183)
Supplement: Supplementary file 1 [file Table_1.DOCX]

**Table S1.** Critical Appraisal of Included Studies

| Study | Target population | participant recruitment | Sample size* | Subject & setting description | Data analysis | Measurement validity* | Measurement reliability* | Statistical analysis | Response rate | **Overall appraisal**** |
| --- | --- | --- | --- | --- | --- | --- | --- | --- | --- | --- |
| Abujilban et al. (2021) | low | high | low | low | unclear | low | low | low | n.a. | **low** |
| Alharbi et al. (2021) | low | high | low | low | unclear | low | low | low | n.a. | **low** |
| Chiaramonte et al. (2021) | low | low | unclear | low | unclear | low | low | low | low | **low** |
| El-Nimr et al. (2021) | low | high | low | low | unclear | high | low | low | n.a. | **low** |
| Hamadani et al. (2020) | high | high | unclear | low | unclear | low | low | high | low | **high** |
| Indu et al. (2021) | low | low | unclear | low | unclear | low | low | high | low | **low** |
| Jetelina et al. (2020) | low | high | unclear | low | unclear | low | low | high | n.a. | **high** |
| Jung et al. (2020) | high | high | unclear | low | unclear | high | low | high | n.a. | **high** |
| Kliem et al. (2021) | low | unclear | unclear | high | unclear | low | low | low | unclear | **high** |
| Lampe et al. (2020) | low | high | low | low | unclear | low | unclear | low | low | **low** |
| Mahmood et al. (2021) | unclear | low | unclear | low | unclear | high | low | low | n.a. | **high** |
| Ojeahere et al. (2021) | high | high | low | low | unclear | high | low | low | n.a. | **high** |
| Pattojoshi et al. (2020) | high | high | low | low | unclear | high | low | high | unclear | **high** |
| Plášilová et al. (2021) | low | low | unclear | low | unclear | unclear | low | low | low | **low** |
| Porter et al. (2021) | low | low | low | low | unclear | unclear | low | low | low | **low** |
| Rashid Soron et al. (2021) | low | high | unclear | low | unclear | high | low | high | n.a. | **high** |
| Sediri et al. (2020) | low | high | unclear | low | unclear | high | low | low | n.a. | **high** |
| Sharma & Khokar (2021) | low | high | unclear | low | unclear | high | low | high | n.a. | **high** |
| Steinhoff et al. (2021) | low | low | low | low | unclear | unclear | low | low | low | **low** |
| Stephenson et al. (2021) | low | low | low | low | unclear | low | low | high | n.a. | **low** |
| Teshome et al. (2021) | unclear | unclear | low | low | unclear | low | unclear | high | unclear | **high** |
| Walsh et al. (2021) | low | high | unclear | low | unclear | low | low | high | low | **low** |

**Note.** Critical appraisal using the JBI critical appraisal checklist for prevalence studies (available at https://jbi.global/critical-appraisal-tools). Studies scored as showing low, unclear, or high risk of bias for each criterion.

* = considered major domain for the current review

** = Overall appraisal of a study to present low risk of bias if at least five of the JBI checklist criteria were fulfilled, including at least one of the three major domains.

n.a. = not applicable
